# *Deinococcus radiodurans* PriA is a Pseudohelicase

**DOI:** 10.1371/journal.pone.0133419

**Published:** 2015-07-16

**Authors:** Matthew E. Lopper, Jacob Boone, Christopher Morrow

**Affiliations:** Department of Chemistry, University of Dayton, Dayton, OH, United States of America; Saint Louis University, UNITED STATES

## Abstract

Reactivation of repaired DNA replication forks in bacteria is catalyzed by PriA helicase. This broadly-conserved bacterial enzyme can remodel the structure of DNA at a repaired DNA replication fork by unwinding small portions of duplex DNA to prepare the fork for replisome reloading. While PriA’s helicase activity is not strictly required for cell viability in *E*. *coli*, the sequence motifs that confer helicase activity upon PriA are well-conserved among sequenced bacterial *priA* genes, suggesting that PriA’s duplex DNA unwinding activity confers a selective advantage upon cells. However, these helicase sequence motifs are not well-conserved among *priA* genes from the Deinococcus-Thermus phylum. Here, we show that PriA from a highly radiation-resistant member of that phylum, *Deinococcus radiodurans*, lacks the ability to hydrolyze ATP and unwind duplex DNA, thus qualifying *D*. *radiodurans* PriA as a pseudohelicase. Despite the lack of helicase activity, *D*. *radiodurans* PriA has retained the DNA binding activity expected of a typical PriA helicase, and we present evidence for a physical interaction between *D*. *radiodurans* PriA and its cognate replicative helicase, DnaB. This suggests that PriA has retained a role in replisome reloading onto repaired DNA replication forks in *D*. *radiodurans* despite its lack of helicase activity.

## Introduction

PriA helicase catalyzes reloading of the DNA replication machinery (the replisome) at repaired DNA replication forks in bacteria, giving bacterial cells the ability to restart DNA replication following disruptive encounters of the replisome with DNA damage [1]. This pathway, known as DNA replication restart, is broadly conserved among diverse bacteria and likely represents an indispensable component of bacterial genome maintenance. Indeed, some bacteria have evolved multiple DNA replication restart pathways that are catalyzed by different sets of primosome proteins and that operate on different types of DNA structures [2, 3]. In *E*. *coli*, a PriA-dependent DNA replication restart pathway catalyzes replisome reloading onto forked DNA structures that lack a gap at the three-way fork junction, while a PriC-dependent DNA replication restart pathway facilitates replisome reloading onto DNA structures that contain a leading strand gap at the fork junction [3]. The presence of multiple DNA replication restart pathways likely confers upon bacterial cells a greater ability to respond to the varied assortment of DNA structures that could be encountered at a stalled or collapsed DNA replication fork [1].

Given the multitude of ways in which DNA can be damaged and the myriad structures that can result from failure of a replisome to fully replicate a chromosome, it seems inevitable that some DNA structures that are created from the premature dissociation of a replisome would require remodeling before a functional replisome could be reloaded [4, 5]. The main DNA replication restart initiator protein, PriA, contains a series of well-conserved helicase motifs that confer upon it the ability to remodel the structure of DNA at a repaired DNA replication fork by unwinding short stretches of duplex DNA at the three-way fork junction [6–8]. This helicase activity requires the coupling of ATP binding and hydrolysis to the translocation of the enzyme along the nucleic acid lattice. Translocation occurs in the 3'-5' direction, which would result in the displacement of a portion of the nascent lagging strand were PriA to unwind duplex DNA at a repaired DNA replication fork [6]. This DNA unwinding activity is thought to create a binding site for reloading the replicative helicase, DnaB, back onto the DNA as part of the fork reactivation process. In addition to unwinding duplex DNA, PriA catalyzes the assembly of additional primosome proteins onto a repaired DNA replication fork. In the PriA-dependent DNA replication restart pathway in *E*. *coli*, these additional primosome proteins include PriB and DnaT [3, 9–12]. The combined activities of PriA, PriB, and DnaT result in reassembly of a functional replisome at a repaired DNA replication fork which allows DNA replication to resume.

**Fig 1:**
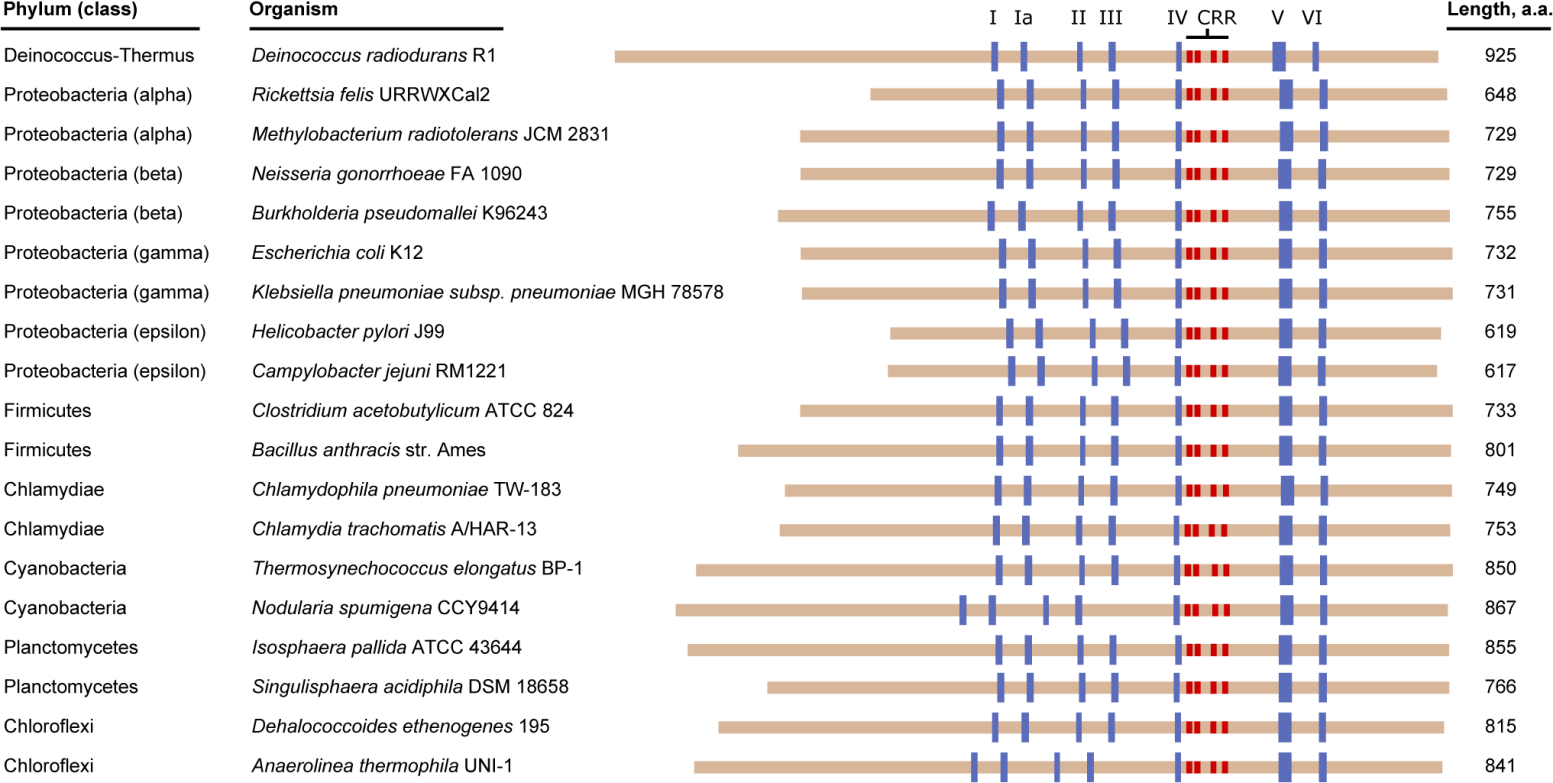
Evolutionary conservation of PriA helicase. The amino acid sequences of PriA helicases from 19 bacterial species representing 7 distinct phyla are aligned with one another and represented by tan bars. The amino terminus of each PriA sequence is oriented at left. Conserved helicase motifs are labeled with roman numerals and are shown as blue boxes, and the cysteine-rich regions (CRR) are shown in red. Note that most of the length variation among PriA helicases maps to the amino-terminal region which contains the DNA binding domain.

In *E*. *coli*, mutations in *priA* have been generated that abolish PriA’s ATPase activity and render it incapable of unwinding duplex DNA, but do not affect its primosome assembly function on model DNA substrates [13]. Interestingly, *E*. *coli* strains carrying these mutant *priA* alleles remain viable and are capable of undergoing recombination, indicating that PriA’s ATPase and duplex DNA unwinding activities are not strictly required for cell survival or assembly of a functional primosome [13–15]. However, despite the fact that PriA’s helicase activity is dispensable under laboratory growth conditions, it seems likely that its helicase activity would play an important genome maintenance role in nature by expanding the cell’s ability to reactivate repaired DNA replication forks on a wider subset of DNA structures, such as those that contain duplex DNA at the three-way junction of a repaired DNA replication fork. This hypothesis is bolstered by the observation that PriA’s helicase motifs are well-conserved among sequenced bacterial *priA* orthologs (Fig 1).

**Fig 2:**
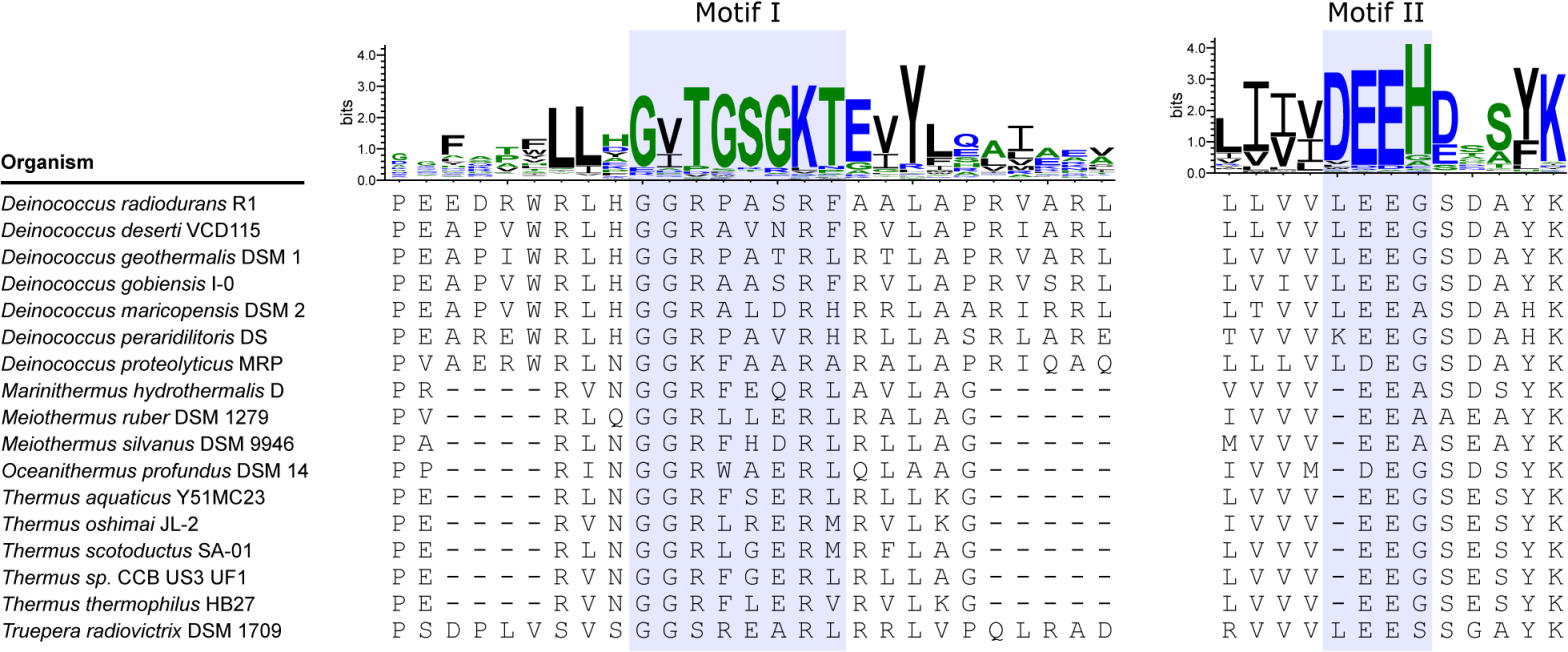
Evolutionary conservation of PriA helicase motifs. Sequence logos were generated for helicase motifs I and II (the Walker A and Walker B boxes, respectively) from a multiple sequence alignment of 100 PriA helicases using the Weblogo 3 sequence logo generator [27, 28]. Each PriA sequence represents a unique genus, and 18 phyla are represented. A multiple sequence alignment of 17 members of the Deinococcus-Thermus phylum appears below each sequence logo. These sequences show significant departure from the otherwise well-conserved helicase motifs.

Members of the Deinococcus-Thermus phylum are notable exceptions to the evolutionary tendency to conserve PriA’s helicase motifs. These bacteria encode a PriA helicase that lacks amino acid residues that are otherwise well-conserved among known bacterial PriAs and are critical for catalyzing ATP hydrolysis and facilitating duplex DNA unwinding. In particular, the Deinococcus-Thermus phylum members have poor matches to the consensus sequences of helicase motifs I and II, also known as the Walker A and Walker B boxes, respectively (Fig 2). In *E*. *coli* PriA and other well-studied helicases, the Walker A box (also known as a phosphate-binding loop, or P-loop) is involved in ATP binding, while the Walker B box is involved in binding an active site Mg^2+^. Both motifs are therefore critical for helicase-catalyzed duplex DNA unwinding [16–18]. Within the Walker A box of Deinococcus-Thermus PriAs, the catalytic lysine residue in the GKT sequence of the motif has been substituted for an arginine residue (Fig 2). This amino acid substitution has been constructed and studied in *E*. *coli* PriA (the K230R PriA variant), and it has been shown to be sufficient to abolish *E*. *coli* PriA’s ATPase and helicase activities [13]. Taken together, these observations led us to hypothesize that Deinococcus-Thermus PriA enzymes would be deficient at hydrolyzing ATP and unwinding duplex DNA, effectively qualifying them as pseudohelicases–enzymes that have clearly evolved from an ancestral helicase but have lost their catalytic activity.

To test this hypothesis, we cloned the *priA* gene from *Deinococcus radiodurans* R1, expressed and purified recombinant *D*. *radiodurans* PriA protein, and tested it for its ability to catalyze ATP hydrolysis and duplex DNA unwinding. Our findings reveal that *D*. *radiodurans* PriA is indeed a pseudohelicase that is unable to hydrolyze ATP or facilitate duplex DNA unwinding. Yet, *D*. *radiodurans* PriA is capable of binding to single-stranded DNA (ssDNA) and fork DNA, and we show evidence for a physical interaction between *D*. *radiodurans* PriA and its cognate replicative helicase, DnaB, using native agarose gel electrophoresis. Collectively, these data suggest that *D*. *radiodurans* PriA has retained its function in DNA replication restart in Deinococcus-Thermus bacteria despite the absence of helicase activity.

## Materials and Methods

### Molecular cloning of *priA* and *dnaB* genes

The *priA* gene of *D*. *radiodurans* was amplified from strain R1 genomic DNA by polymerase chain reaction (PCR) using primers 5'-GCG TAT TCC ATA TGA CTC TCT GGA CCG TTT C and 5'-GTC ACG GAT CCT CAG GTC GAG CGC GGG TTC AC. The PCR-amplified product was cloned into the pET28b expression vector (Novagen) using NdeI and BamHI restriction sites. The resulting plasmid contains a six-histidine tag and thrombin cleavage site fused to the 5' end of *D*. *radiodurans priA*. The *dnaB* gene of *D*. *radiodurans* was amplified from strain R1 genomic DNA by polymerase chain reaction (PCR) using primers 5'-GCG TAT TCC ATA TGG AAA CGA CTC CGC GTG TG and 5'-GTC ACG GAT CCT CAC ATC CCC TCC GGC GCC. The PCR-amplified product was cloned into the pET28b expression vector (Novagen) using NdeI and BamHI restriction sites. The resulting plasmid contains a six-histidine tag and thrombin cleavage site fused to the 5' end of *D*. *radiodurans dnaB*. The fidelity of the cloned genes was confirmed by DNA sequencing. The *priA* gene of *E*. *coli* strain K12 was cloned as previously described [19].

### Purification of PriA and DnaB proteins

*D*. *radiodurans* PriA protein was purified from BL21(DE3) *E*. *coli* harboring the pET28b:*Drad-priA* plasmid. Cells were grown in Luria Bertani (LB) medium containing 50 μg·mL^-1^ kanamycin at 37°C until an OD_600_ of 0.4–0.6 was reached. Expression of *D*. *radiodurans* PriA protein was induced with 0.5 mM isopropyl β-D-1-thiogalactopyranoside (IPTG) for 4 h and cells were harvested by centrifugation at 5,000 × *g*. Cells were lysed in 10 mM Tris·HCl pH 8.5, 10% (v/v) glycerol, 0.5 M NaCl, 10 mM imidazole, 1 mM 2-mercaptoethanol, 1 mM phenylmethylsulfonyl fluoride (PMSF) by sonication on ice. The lysate was clarified by centrifugation at 40,000 × *g*. His-tagged *D*. *radiodurans* PriA was bound to nickel-NTA agarose (Qiagen) and eluted in 10 mM Tris·HCl pH 8.5, 10% (v/v) glycerol, 0.1 M NaCl, 250 mM imidazole, 1 mM 2-mercaptoethanol. The nickel-NTA agarose eluate was dialyzed against 10 mM Tris·HCl pH 8.5, 10% (v/v) glycerol, 0.1 M NaCl, 1 mM 2-mercaptoethanol and incubated with thrombin to remove the His-tag, leaving a Gly-Ser-His sequence at the amino-terminus directly preceding the first methionine residue. Thrombin-cleaved *D*. *radiodurans* PriA was applied to a HiPrep 16/10 Sepharose QFF ion exchange chromatography column (GE Healthcare) and the column was resolved using a linear gradient of NaCl from 0.1 M to 1 M in 10 mM Tris·HCl pH 8.5, 10% (v/v) glycerol, 1 mM 2-mercaptoethanol. *D*. *radiodurans* PriA fractions were pooled, concentrated to less than 2 mL, and resolved through a HiPrep HR 16/10 Sephacryl S-300 size exclusion chromatography column (GE Healthcare) in 10 mM Tris·HCl pH 8.5, 10% (v/v) glycerol, 0.5 M NaCl, 1 mM 2-mercaptoethanol at a flow rate of 0.5 mL/min. *D*. *radiodurans* PriA fractions were pooled, concentrated, and stored at -80°C. The recombinant *E*. *coli* PriA protein was purified as previously described [19].

*D*. *radiodurans* DnaB protein was purified from BL21(DE3) *E*. *coli* harboring the pET28b:*Drad-dnaB* plasmid. Cells were grown in LB medium containing 50 μg·mL^-1^ kanamycin at 37°C until an OD_600_ of 0.4–0.6 was reached. Expression of *D*. *radiodurans* DnaB protein was induced with 0.5 mM IPTG for 4 h and cells were harvested by centrifugation at 5,000 × *g*. Cells were lysed in 10 mM Tris·HCl pH 8.5, 10% (v/v) glycerol, 0.5 M NaCl, 10 mM imidazole, 1 mM 2-mercaptoethanol, 1 mM PMSF by sonication on ice. The lysate was clarified by centrifugation at 40,000 × *g*. His-tagged *D*. *radiodurans* DnaB was bound to nickel-NTA agarose (Qiagen) and eluted in 10 mM Tris·HCl pH 8.5, 10% (v/v) glycerol, 0.1 M NaCl, 250 mM imidazole, 1 mM 2-mercaptoethanol. The nickel-NTA agarose eluate was dialyzed against 10 mM Tris·HCl pH 8.5, 10% (v/v) glycerol, 0.1 M NaCl, 1 mM 2-mercaptoethanol and incubated with thrombin to remove the His-tag, leaving a Gly-Ser-His sequence at the amino-terminus directly preceding the first methionine residue. Thrombin-cleaved *D*. *radiodurans* DnaB was resolved through a HiPrep HR 16/10 Sephacryl S-300 size exclusion chromatography column (GE Healthcare) in 10 mM Tris·HCl pH 8.5, 10% (v/v) glycerol, 0.5 M NaCl, 1 mM 2-mercaptoethanol at a flow rate of 0.5 mL/min. *D*. *radiodurans* DnaB fractions were pooled, concentrated, and stored at -80°C.

The concentrations of recombinant proteins were determined by measuring their absorbance at 280 nm in 6.0 M guanidine-HCl using molar extinction coefficients of 184,370 M^-1^·cm^-1^ for *D*. *radiodurans* PriA, 12,920 M^-1^·cm^-1^ for *D*. *radiodurans* DnaB, and 105,870 M^-1^·cm^-1^ for *E*. *coli* PriA.

### Nucleic acids

For the DNA unwinding assays and DNA binding assays, a fully-duplex DNA fork substrate with a 25 bp parental duplex arm, a 25 bp nascent leading strand arm, and a 25 bp 3'-fluorescein-labeled nascent lagging strand arm was constructed by annealing the following partially complementary DNA oligonucleotides: (template leading strand) 5'-GTC GGA TCC TCT AGA CAG CTC CAT GAT CAC TGG CAC TGG TAG AAT TCG GC; (template lagging strand) 5'-AAC GTC ATA GAC GAT TAC ATT GCT ACA TGG AGC TGT CTA GAG GAT CCG AC; (nascent leading strand) 5'-GCC GAA TTC TAC CAG TGC CAG TGA T; (nascent lagging strand with 3' fluorescein) 5'-TAG CAA TGT AAT CGT CTA TGA CGT T. The oligonucleotides were annealed in 10 mM Tris·HCl pH 8, 50 mM NaCl, 1 mM EDTA at a 2:1 molar ratio of non-labeled DNA to fluorescein-labeled DNA. The DNAs were incubated at 95°C for 5 min, slow-cooled to 70°C and incubated at that temperature for 60 min, and slow-cooled to 25°C. The duplex DNA was gel-purified through a 6% polyacrylamide gel using 100 mM Tris·borate pH 8.3, 2 mM EDTA as the electrophoresis buffer. The DNA was excised from the polyacrylamide gel, electroeluted using the same electrophoresis buffer, dialyzed against 10 mM Tris·HCl pH 8, 5 mM MgCl_2_, aliquoted, and stored at -20°C. An 18-base mixed-sequence ssDNA oligonucleotide (5'-AAG CAC AAT TAC CCA CGC) labeled at its 3' end with fluorescein was also used for the equilibrium DNA binding assays. A homopolymer dT_36_ ssDNA oligonucleotide was used for steady-state enzyme kinetics experiments to measure DNA-dependent rates of PriA-catalyzed ATP hydrolysis. All oligonucleotides were purchased from Integrated DNA technologies, Inc (Coralville, IA).

### DNA unwinding assays

The DNA fork substrate was diluted to 1 nM in 20 mM Tris·HCl pH 8, 50 mM NaCl, 3 mM MgCl_2_, 1 mM 2-mercaptoethanol, 1 mM ATP. Indicated concentrations of *D*. *radiodurans* PriA or *E*. *coli* PriA were added to the reaction mixtures and incubated at 37°C for 10 min to facilitate duplex DNA unwinding. Reactions were stopped by addition of SDS to a final concentration of 1% (w/v). The amount of duplex DNA unwound was determined by measuring the fluorescence anisotropy of the samples after addition of SDS using a Beacon 2000 fluorescence polarization system (Invitrogen). Fluorescence anisotropy values were compared to the fluorescence anisotropy of the DNA fork substrate incubated in buffer alone (representing the fully intact DNA fork substrate) and the fluorescence anisotropy of the DNA fork substrate after being heated to 95°C and rapidly cooled to 25°C (representing the fully denatured DNA fork substrate) to calculate the fraction of DNA unwound.

### Equilibrium DNA binding assays

Serial dilutions of *D*. *radiodurans* PriA or *E*. *coli* PriA were incubated with 1 nM fluorescein-labeled 18-mer ssDNA oligonucleotide or 1 nM fluorescein-labeled fork DNA in 20 mM Tris·HCl pH 8, 15% (v/v) glycerol, 50 mM NaCl, 1 mM MgCl_2_, 1 mM 2-mercaptoethanol, 0.1 g·L^-1^ bovine serum albumin (BSA), and the fluorescence anisotropy of the mixtures was measured at 25°C with a Beacon 2000 fluorescence polarization system. The fluorescence anisotropy data were used to calculate the fraction of the fluorescein-labeled DNA bound as a function of total PriA concentration by using the fluorescence anisotropy of the fluorescein-labeled DNA alone as the unbound DNA control and the fluorescence anisotropy of the fluorescein-labeled DNA in the presence of the highest concentration of PriA tested as the fully-bound DNA control. The fraction of DNA bound as a function of PriA concentration was modeled using a four-parameter sigmoidal growth function [y = (a·b + c·x^d^)/(b + x^d^)] where a is the lower asymptote (corresponding to the fluorescence anisotropy of the unbound DNA), c is the upper asymptote (corresponding to the fluorescence anisotropy of the fully-bound DNA), and b and d influence the shape of the sigmoid (CurveExpert 1.3). Apparent dissociation constants were determined by interpolating the concentration of PriA necessary to bind one-half of the fluorescein-labeled DNA.

### ATP hydrolysis assays

PriA-catalyzed ATP hydrolysis was measured using a coupled spectrophotometric assay. This assay uses an ATP regeneration system that converts ADP to ATP in a reaction that is coupled to the oxidation of NADH to NAD^+^ [20]. The coupled reaction can be monitored spectrophotometrically by measuring the decrease in absorbance at 340 nm due to NADH oxidation. Concentrations of *D*. *radiodurans* PriA or *E*. *coli* PriA ranging from 0–100 nM were incubated with 1 μM dT_36_ and 1 mM ATP in 20 mM Hepes pH 8, 50 mM NaCl, 7 mM 2-mercaptoethanol, 2 mM phosphoenolpyruvate, 0.1 mM NADH, 7 units·mL^-1^ pyruvate kinase, 10 units·mL^-1^ lactate dehydrogenase, 0.1 g·L^-1^ BSA at 37°C. Steady-state Δ[NADH]/Δt rates were calculated using the molar extinction coefficient 6,220 M^-1^·cm^-1^ for NADH, and these rates are equivalent to Δ[ATP]/Δt.

### Native agarose gel electrophoresis

Indicated amounts of *D*. *radiodurans* PriA and *D*. *radiodurans* DnaB were mixed and incubated on ice for 10 min prior to the addition of gel loading buffer consisting of 20% (v/v) glycerol, 0.2% (w/v) bromophenol blue, 0.12 M Tris base. Samples were added to wells of horizontal 0.6% (w/v) agarose gels that were prepared with 25 mM Tris·HCl pH 8.5 and 19.2 mM glycine. We obtained the best results when the wells were cast in the middle of the gel as opposed to one end. The gels were submerged in the same buffer solution and the samples were electrophoresed at 50 V for approximately 4 h at 4°C. The gels were stained with 0.12% (w/v) Coomassie Brilliant Blue R-250 in 45% (v/v) methanol and 10% (v/v) acetic acid and destained with 45% (v/v) methanol and 10% (v/v) acetic acid.

### Protein unfolding assay

*D*. *radiodurans* PriA at a final concentration of 0.1 μM was incubated with 0–6 M guanidine-HCl in a buffered solution consisting of 50 mM Hepes pH 7 and 1 mM 2-mercaptoethanol at 25°C. Fluorescence measurements were made at 25°C using a Cary Eclipse fluorescence spectrophotometer using an excitation wavelength of 280 nm and a 5 nm excitation filter slit width. Emission was recorded from 300 nm – 400 nm using a 5 nm emission filter slit width and a scan rate of 30 nm·min^-1^. Spectra for the protein samples were baseline subtracted using the spectra of the buffered solutions without PriA at each concentration of guanidine-HCl. The fluorescence intensities at 324 nm were used to calculate the fraction of *D*. *radiodurans* PriA folded at each concentration of guanidine-HCl, where the 6 M guanidine-HCl sample represents the unfolded spectrum and the sample with no guanidine-HCl represents the folded spectrum [21]. The fraction of PriA folded as a function of guanidine-HCl was modeled using a four-parameter function [y = (a + b·x)/(1 + c·x + d·x^2^)] where a is the upper asymptote (corresponding to the folded protein), c is the lower asymptote (corresponding to the unfolded protein), and b and d influence the shape of the curve (CurveExpert 1.3). This model was used to interpolate the concentration of denaturant at which one-half of the protein is unfolded, [D]_1/2_.

## Results and Discussion

**Fig 3:**
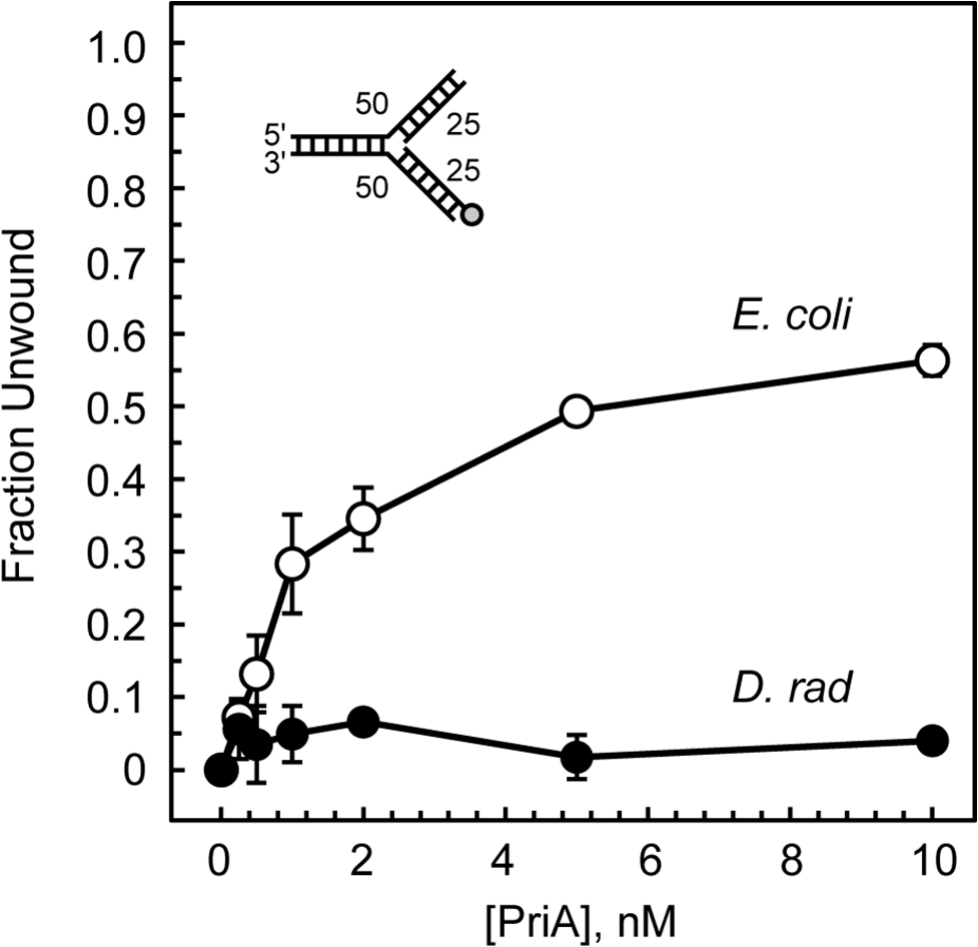
PriA-catalyzed duplex DNA unwinding. *D*. *radiodurans* PriA (closed circles) and *E*. *coli* PriA (open circles) were individually tested for their ability to catalyze unwinding of a forked DNA substrate with a 25 bp fluorescently-labeled nascent lagging strand arm as described in Materials and Methods. Data are reported in triplicate and error bars represent one standard deviation of the mean.

To test the hypothesis that *D*. *radiodurans* PriA is a pseudohelicase, we cloned the *D*. *radiodurans priA* gene, expressed the recombinant PriA protein in an *E*. *coli* host, and purified the PriA protein to approximately 95% purity as estimated by SDS-PAGE analysis and Coomassie Brilliant Blue-R250 staining (data not shown). To determine if *D*. *radiodurans* PriA has helicase activity, we employed a fluorescence anisotropy-based duplex DNA unwinding assay using a fork DNA with a fluorescein-labeled 25 bp nascent lagging strand arm as the substrate, as described previously [22]. In brief, the fluorescein-labeled fork DNA substrate was incubated with either *E*. *coli* PriA or *D*. *radiodurans* PriA in the presence of ATP for 10 min at 37°C, the reactions were terminated by addition of SDS, and the fluorescence anisotropy of the samples was measured. The degree of unwinding of the fork DNA substrate was determined by comparing the fluorescence anisotropy of the samples to that of the fork DNA substrate incubated in buffer alone (representing the fully intact fork DNA substrate) and to samples heated briefly to 95°C and fast-cooled back to 25°C (representing the fully denatured fork DNA substrate). This allowed us to measure the fraction of the fork DNA substrate that is unwound by various concentrations of the PriA proteins. As expected, *E*. *coli* PriA is capable of unwinding the fork DNA substrate in a PriA-dependent manner with 56% unwinding of the fork DNA occurring at 10 nM PriA (Fig 3). *D*. *radiodurans* PriA shows negligible levels of unwinding on the same fork DNA substrate. At 10 nM *D*. *radiodurans* PriA, only 4% of the fork DNA is unwound (Fig 3).

**Fig 4:**
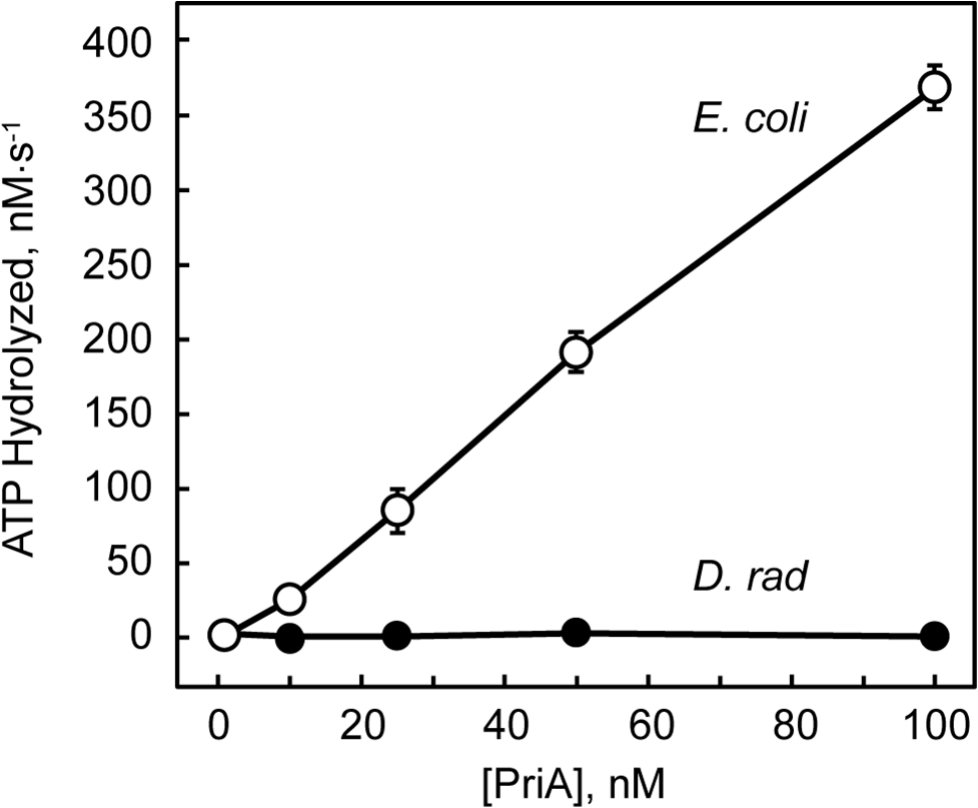
PriA-catalyzed ATP hydrolysis. *D*. *radiodurans* PriA (closed circles) and *E*. *coli* PriA (open circles) were individually tested for their ability to catalyze hydrolysis of ATP in the presence of a 36 base dT homopolymer as described in Materials and Methods. Data are reported in triplicate and error bars represent one standard deviation of the mean.

Given the lack of conservation of catalytic amino acid residues in the Walker A and Walker B boxes of *D*. *radiodurans* PriA, it seems likely that the inability of *D*. *radiodurans* PriA to unwind the fork DNA substrate in the duplex DNA unwinding assays stems from an inability of *D*. *radiodurans* PriA to hydrolyze ATP. Therefore, we measured the ATPase activity of *D*. *radiodurans* PriA using a coupled spectrophotometric assay that relates ATP hydrolysis to the oxidation of NADH to NAD^+^. We then compared the ATPase activity of *D*. *radiodurans* PriA to that of *E*. *coli* PriA. This assay uses a homopolymer dT_36_ oligonucleotide to stimulate the ATPase activity of PriA since PriA’s ability to catalyze ATP hydrolysis is known to be strongly dependent upon the presence of a DNA lattice [22, 23]. Under these experimental conditions, 100 nM *E*. *coli* PriA catalyzes ATP hydrolysis at a rate of 369 ± 9 nM ATP·sec^-1^ (Fig 4). Under these same conditions, *D*. *radiodurans* PriA does not catalyze ATP hydrolysis at a measurable rate (Fig 4).

**Fig 5:**
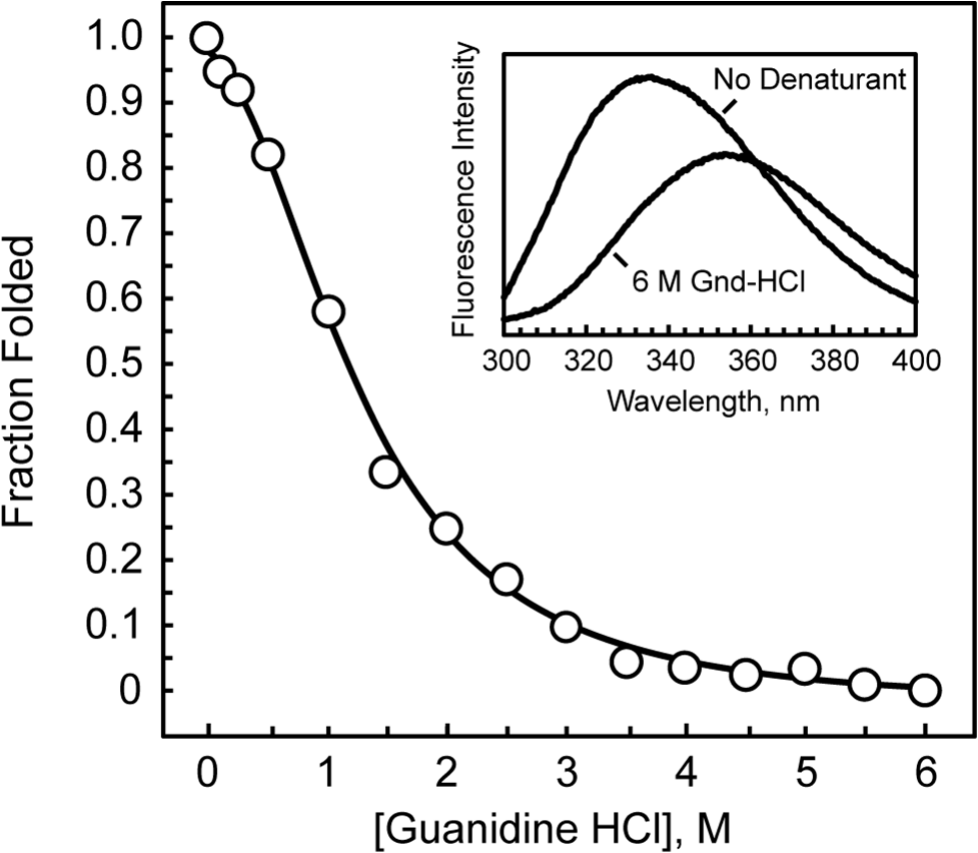
Guanidine-mediated unfolding of PriA. *D*. *radiodurans* PriA was incubated with indicated concentrations of guanidine-HCl and PriA’s intrinsic fluorescence was measured as described in Materials and Methods. The inset shows the fluorescence emission spectrum for *D*. *radiodurans* PriA in the presence and absence of 6 M guanidine-HCl. The best fit curve for the plot of the fraction of PriA folded as a function of guanidine-HCl was generated by fitting the data to a four-parameter function [y = (a + b·x)/(1 + c·x + d·x^2^)] (CurveExpert 1.3). Data are reported for one representative experiment.

To verify that our recombinant *D*. *radiodurans* PriA protein is not unfolded, we measured its intrinsic fluorescence as a function of increasing concentrations of the denaturing agent, guanidine-HCl. The fluorescence emission spectra of *D*. *radiodurans* PriA in the presence and absence of 6 M guanidine-HCl clearly differ from one another (Fig 5). The spectra show the greatest difference in fluorescence intensity at 324 nm, so fluorescence intensity measurements at this wavelength were used to calculate the fraction of PriA folded at various concentrations of denaturant. The resulting unfolding curve lacks a strong folded-state baseline (Fig 5), which precludes an estimation of the free energy of the unfolding reaction. The denaturant concentration at the midpoint of the unfolding curve, [D]_1/2_, is 1.2 M for guanidine-HCl-induced unfolding. Therefore, regardless of the mechanism by which PriA unfolds or the presence and number of any unfolding intermediates, these data indicate that the recombinant *D*. *radiodurans* PriA protein that we expressed in *E*. *coli*, purified, and tested for duplex DNA unwinding activity, DNA binding activity, and ATPase activity is not unfolded.

**Fig 6:**
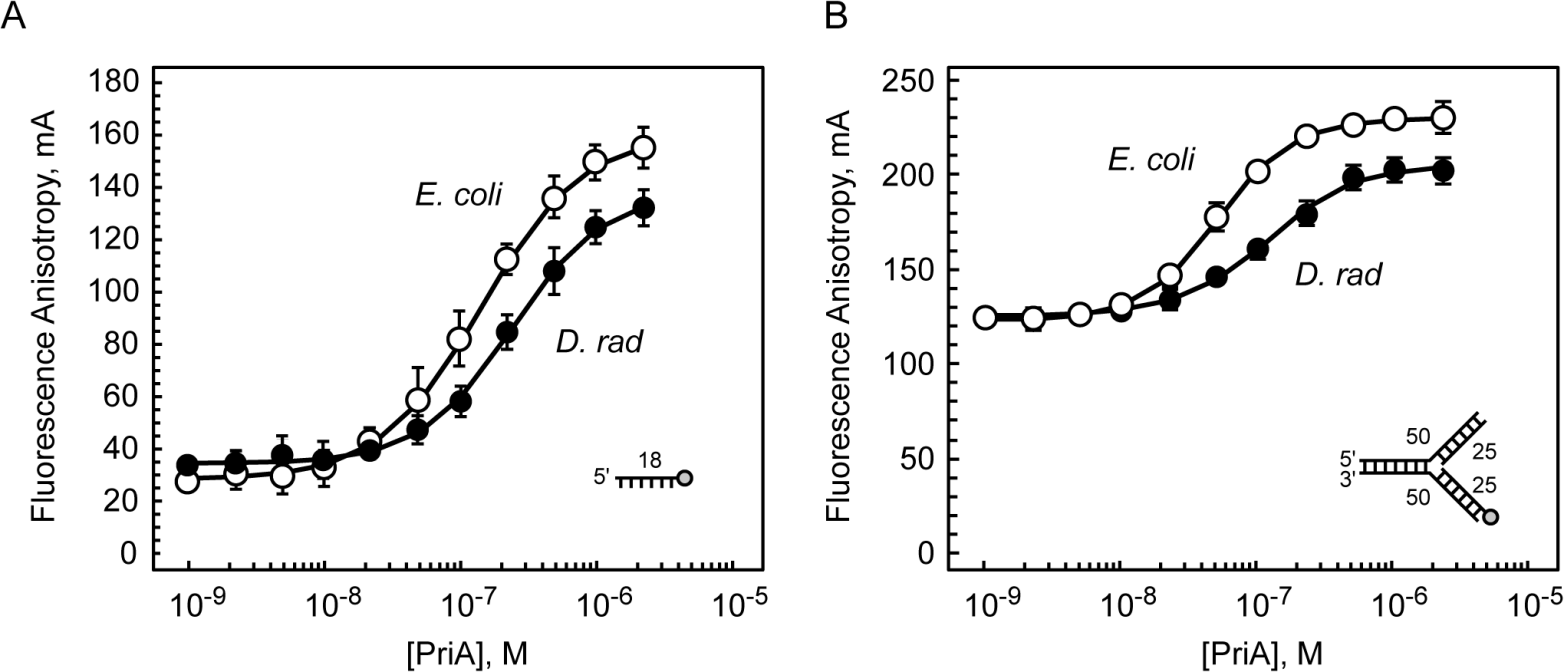
DNA binding activity of PriA. *D*. *radiodurans* PriA (closed circles) and *E*. *coli* PriA (open circles) were individually tested for their ability to bind to A) an 18-base ssDNA oligonucleotide, or B) a fully-duplex fork DNA with 25 bp arms using a fluorescence-anisotropy-based equilibrium DNA binding assay as described in Materials and Methods. Data are reported in triplicate and error bars represent one standard deviation of the mean.

Given the lack of helicase activity, we sought to determine if *D*. *radiodurans* PriA possesses any other activities that would be expected for a functional PriA. Thus, we tested for its ability to bind to an 18-base mixed-sequence ssDNA oligonucleotide using a fluorescence anisotropy-based equilibrium DNA binding assay. For these experiments, *D*. *radiodurans* PriA at various concentrations was incubated with a fixed concentration of fluorescein-labeled ssDNA and the fluorescence anisotropy of the mixtures was measured. We observed a *D*. *radiodurans* PriA-dependent increase in the fluorescence anisotropy of the fluorescein-labeled ssDNA, indicating that *D*. *radiodurans* PriA is capable of binding to ssDNA (Fig 6A). These equilibrium DNA binding assays were also conducted using *E*. *coli* PriA and the same ssDNA substrate. As expected, we observed an *E*. *coli* PriA-dependent increase in the fluorescence anisotropy of the fluorescein-labeled ssDNA, confirming many previously published reports that *E*. *coli* PriA binds ssDNA (Fig 6A) [24–26]. The calculated apparent dissociation constants are 129.2 ± 11.9 nM for the *E*. *coli* PriA-ssDNA interaction, and 215.4 ± 17.0 nM for the *D*. *radiodurans* PriA-ssDNA interaction. These apparent dissociation constants differ only slightly from one another, suggesting that the structural features of PriA that are required for binding to ssDNA have been largely conserved between these two PriAs throughout evolution. This also indicates that the lack of ATPase activity observed for *D*. *radiodurans* PriA is not due to a defect in binding ssDNA.

We next tested the ability of *D*. *radiodurans* PriA to bind to a fork DNA substrate which mimics the type of DNA structure that PriA would presumably bind *in vivo* during replisome reactivation. As with the ssDNA substrate, we observed a *D*. *radiodurans* PriA-dependent increase in the fluorescence anisotropy of the fluorescein-labeled fork DNA, indicating that *D*. *radiodurans* PriA is capable of binding to a fork DNA structure (Fig 6B). Likewise, *E*. *coli* PriA binds to the same fork DNA substrate and does so with an affinity that is similar to that of *D*. *radiodurans* PriA (Fig 6B). The calculated apparent dissociation constants are 52.1 ± 1.0 nM for the *E*. *coli* PriA-fork DNA interaction, and 108.5 ± 1.7 nM for the *D*. *radiodurans* PriA-fork DNA interaction. We have previously used this same equilibrium DNA binding assay and the same fork DNA substrate to measure the interaction between *Neisseria gonorrhoeae* PriA and fork DNA and determined the apparent dissociation constant to be 134 ± 22 nM for the *N*. *gonorrhoeae* PriA-fork DNA interaction [22]. Thus, the affinity of *D*. *radiodurans* PriA for the fork DNA substrate lies between that of the *E*. *coli* and *N*. *gonorrhoeae* PriAs, both of which have well-documented helicase activities.

**Fig 7:**
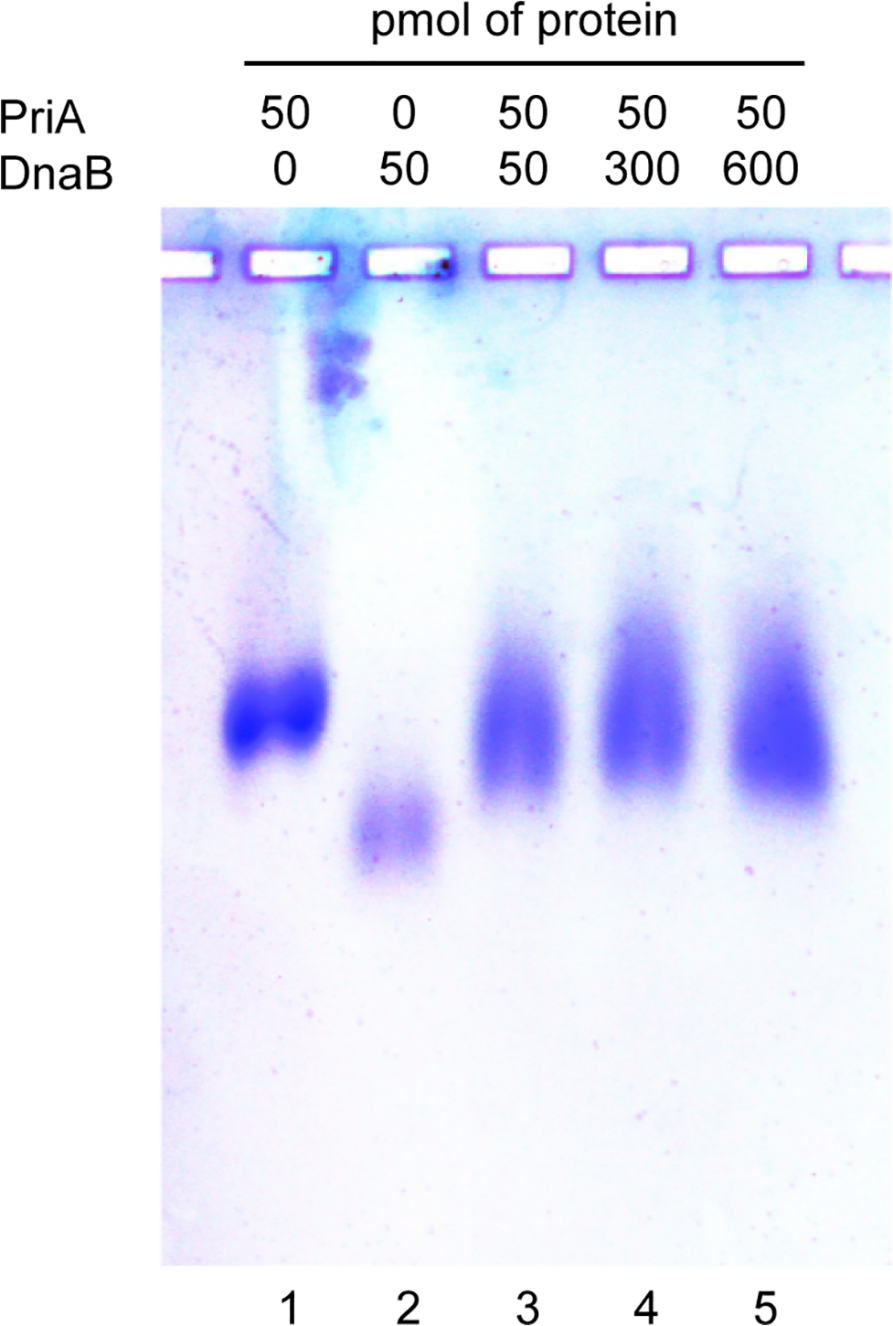
Physical interaction of *D. radiodurans* PriA and *D. radiodurans* DnaB. Indicated amounts of *D*. *radiodurans* PriA and *D*. *radiodurans* DnaB were incubated either alone or together and electrophoresed through an agarose gel under native conditions as described in Materials and Methods.

Given that *D*. *radiodurans* PriA binds ssDNA and fork DNA, we next wanted to determine if it physically interacts with any other proteins that would be expected to be present at a repaired DNA replication fork in the context of a replisome reloading pathway. Since *D*. *radiodurans* lacks clear homologs of *priB* and *dnaT* in its genome, we tested for the ability of *D*. *radiodurans* PriA to bind to its cognate replicative helicase, DnaB. While a PriA-DnaB physical interaction has not been reported in the well-studied *E*. *coli* system, it is likely that *E*. *coli* PriA leads to the recruitment of DnaB to a repaired replication fork through some type of physical interaction, perhaps mediated indirectly through other primosome proteins with which PriA physically interacts. We used native agarose gel electrophoresis to test for a physical interaction between *D*. *radiodurans* PriA and DnaB proteins. When 50 pmol of *D*. *radiodurans* PriA is resolved by itself through an agarose gel under native conditions, Coomassie Brilliant Blue R250 staining reveals the presence of a single band (Fig 7, lane 1). Likewise, resolution of 50 pmol of *D*. *radiodurans* DnaB by itself under the same electrophoresis conditions produces a single band (Fig 7, lane 2). These individual PriA and DnaB bands do not comigrate through the gel. However, when the PriA and DnaB proteins are mixed together and the mixture is resolved through a native agarose gel, only a single band is observed (Fig 7, lanes 3–5). This band migrates approximately midway between the positions of free PriA and free DnaB in the gel, suggesting that it represents a complex of the PriA and DnaB proteins.

In conclusion, we have demonstrated that PriA from *D*. *radiodurans* is a pseudohelicase that has retained the DNA binding activity of a typical bacterial PriA but can neither hydrolyze ATP nor unwind duplex DNA. We have also shown evidence for a physical interaction between *D*. *radiodurans* PriA and its cognate replicative helicase, DnaB. Collectively, these data suggest that *D*. *radiodurans* PriA has retained a function in DNA replication restart pathways despite its inability to unwind duplex DNA at a repaired DNA replication fork. Whether *D*. *radiodurans* uses another DNA helicase for this function, or catalyzes replisome reloading onto DNA structures that do not require remodeling by a helicase remains unclear.
